# Real-time detection of somatic hybrid cells during electrofusion of carrot protoplasts with stably labelled mitochondria

**DOI:** 10.1038/s41598-020-75983-w

**Published:** 2020-11-02

**Authors:** Miron Gieniec, Julianna Siwek, Tomasz Oleszkiewicz, Katarzyna Maćkowska, Magdalena Klimek-Chodacka, Ewa Grzebelus, Rafal Baranski

**Affiliations:** Department of Plant Biology and Biotechnology, Faculty of Biotechnology and Horticulture, University of Agriculture in Krakow, AL. 29 Listopada 54, 31-425 Krakow, Poland

**Keywords:** Cell culture, Fluorescence imaging, Genetic hybridization

## Abstract

Somatic hybridisation in the carrot, as in other plant species, enables the development of novel plants with unique characteristics. This process can be induced by the application of electric current to isolated protoplasts, but such electrofusion requires an effective hybrid cell identification method. This paper describes the non-toxic fluorescent protein (FP) tagging of protoplasts which allows discrimination of fusion components and identification of hybrids in real-time during electrofusion. One of four FPs: cyan (eCFP), green (sGFP), yellow (eYFP) or the mCherry variant of red FP (RFP), with a fused mitochondrial targeting sequence, was introduced to carrot cell lines of three varieties using *Agrobacterium*-mediated transformation. After selection, a set of carrot callus lines with either GFP, YFP or RFP-labelled mitochondria that showed stable fluorescence served as protoplast sources. Various combinations of direct current (DC) parameters on protoplast integrity and their ability to form hybrid cells were assessed during electrofusion. The protoplast response and hybrid cell formation depended on DC voltage and pulse time, and varied among protoplast sources. Heterofusants (GFP + RFP or YFP + RFP) were identified by detection of a dual-colour fluorescence. This approach enabled, for the first time, a comprehensive assessment of the carrot protoplast response to the applied electric field conditions as well as identification of the DC parameters suitable for hybrid formation, and an estimation of the electrofusion success rate by performing real-time observations of protoplast fluorescence.

## Introduction

Somatic hybridisation, also called protoplast or somatic cell fusion, is a process during which protoplasts, usually isolated from different plant species or genera, are forced to merge^1^. Homo- or heterofusants are formed in a homo- or heterofusion process, respectively, depending on whether the merging protoplasts possess the same genetic material or not^2^. Methods of somatic hybridisation have been known for almost 50 years since the first report, published in 1972, presented the mechanical fusion between *Nicotiana glauca* and *N. langsdorffii* protoplasts^3^. Since then, stimulation of somatic hybridisation has been a valuable approach used in plant genetic engineering and breeding. It is one of the few methods that can bypass the pre- and post-zygotic barriers occurring in the process of sexual plant interbreeding^4–6^. The method also supports the use of wild relatives as a source of broad variation and the introduction of new characteristics present in a gene pool to cultivated forms^7–9^. Thus, somatic hybridisation has been used for intra- and inter-specific hybrid development^1^. In addition, intergeneric hybrids have also been obtained, such as in case of protoplasts isolated from the Solanaceae, Brassicaceae and Rutaceae plants^8–11^. Somatic hybrids may exhibit unique and useful characteristics derived from the combination of two parental components. Hence, novel plants with altered phenotypes and increased tolerance to biotic and abiotic stress have been created^12–14^. Only a few reports concern somatic hybridisation in the carrot. Up to now, this technique has been used to create carrot hybrids with *N. tabacum*^15^, *Oryza sativa*^16^, *Hordeum vulgare*^17^, *Panax quinquefolius*^18^, a wild carrot relative *Daucus capillifolius*^19,20^ and various cultivated carrot accessions^21^. These works were aimed towards the development of plants showing tolerance to abiotic factors, such as cold and salinity, the transfer of cytoplasmic male sterility (CMS) for facilitating F1 hybrid seed production and enhancing desirable culinary, nutritional or processing traits^18,21,22^.

The essential steps in hybrid development are: (1) isolation of protoplasts, (2) fusion, (3) hybrid cell identification and selection and (4) regeneration and plant growth. Validation of the cell, tissue or plant hybrid status could be required at any stage^23^. The known selection strategies include genetic or physiological complementation, inactivation of parental cells, or mechanical selection (based on the protoplast morphological differences or their differential staining) using a micromanipulator or cell sorter^24^. However, due to the specificity of individual species and the difficulties in developing mutants for selection based on complementation, the verification systems for hybrid identification must be established individually. There are still several limitations to the somatic hybridisation technique and, commonly, the most critical are (1) the lack of simple and fast methods for real-time detection and discrimination of somatic hybrids immediately after the fusion and (2) the low efficiency of somatic hybridisation^25^. The fusion of two protoplasts is possible when both components stay in contact and their cell membranes adhere to each other. Polyethylene glycol (PEG) is the most common chemical fusogen used to mediate protoplast hybridisation^26^. PEG molecules induce agglutination of protoplasts, reversible membrane breakdown and fusion. The commonly applied 40–55% PEG solutions are non-toxic to protoplasts and can be used for a wide range of plant species. However, an irreversible membrane breakdown leading to protoplast damage is very often observed, and it drastically decreases fusion efficiency^27^. Therefore, in recent years, electric current-mediated fusion has become an alternative approach due to the considerable advantages of electrofusion over chemically induced fusion, including easy operation, low toxicity and applicability to a wide range of cell types^28^. The application of electric current, instead of chemical inducers, prevents the loss of protoplasts and hybrids that commonly occurs during cell washing of fusogens, so the fusion efficiency may be higher^27^.

During electrofusion, the contact between the protoplasts is obtained via the application of alternating current that causes the protoplasts to set up in characteristic pearl chains^29–31^. Then, pulses of direct current (DC) cause reversible cell membrane disintegration and pore formation, finally leading to the protoplast fusion. Successful electrofusion requires the optimisation of the electric current parameters that is usually laborious and time-consuming, mainly because the hybrid cell is often indistinguishable from the parental cells when observed either during the hybridisation or immediately after the fusion^32^. Hence, the identification of hybrids and estimation of fusion efficiency require a long-lasting culture, plant development and evaluation with regard to new phenotypic characters. The use of various molecular markers or chromosome analyses can also be utilised when hybrid tissue or the whole plant is obtained, thus they do not allow hybrid identification during the fusion or just after its completion^9,33,34^.

The labelling of protoplasts is advantageous when identification at early steps of hybridisation is required. For this purpose, isolated protoplasts are treated with fluorochromes emitting green fluorescence (such as fluorescein diacetate—FDA or fluorescein isothiocyanate—FITC) or red fluorescence (rhodamine B thiocyanate—RBITC or tetramethylrhodamine isothiocyanate—TRITC). Such vital staining allows rapid protoplast discrimination, and, moreover, hybrid cells can be identified due to the emission of dual-colour fluorescence^35^. This system facilitates the appraisal of the rate of heterofusion, although it has considerable limitations. Some dyes can be toxic to humans, treatment of isolated protoplasts with chemicals reduces the number of viable protoplasts, fluorochromes are prone to photo-bleaching and fluorescence emission is transient. Furthermore, dye leakage occurs when cell membranes are treated with electric pulses. Hence, protoplasts and, as a consequence, hybrids are labelled only temporarily, meaning that observations must be performed very quickly^36,37^.

The above-listed problems can be mostly avoided when the protoplasts are stably labelled with fluorescent proteins (FPs) enabling real-time detection. These proteins contain a chromophore usually composed of three amino acids which form a ring system with conjugated double bonds. The excitation of this system by light at a specific wavelength causes chromophore fluorescence, and, as a result, the emission of light in a longer wavelength is observed^38,39^. FPs are small and non-toxic proteins and their detection through visual fluorescence observation can be easily performed using a hand-held lamp or a microscope equipped with filters ensuring correct excitation and emission wavelengths that fit the characteristics of the particular fluorescent protein variant^40^. Furthermore, fluorescence can be observed in living cells at any stage without the need for any exogenous substrates^41^. They are thus convenient markers, enabling cell and organelle labelling^42^. The genes coding for FPs have been introduced to many organisms using genetic transformation techniques, and the transformants show fluorescence after being exposed to light whose spectrum could be absorbed by the newly synthesised FP in the cells^43–45^. The green fluorescent protein (GFP) and its variants are the most common FPs introduced to plants^46^. GFP has also been expressed in carrot protoplasts, cells, callus, and plant organs^47–49^. GFP has been used in somatic hybridisation for continuous monitoring of the fusion process and hybrid plant development in citrus^24^, the selection of hybrid callus lines after protoplast electrofusion in orange^50^, the hybrid callus lines and embryoid formation in citrus^51^ and the mitochondrial fusion in plants^52^.

A number of other FPs emitting fluorescence in non-overlapping spectral ranges are available now^53^. They can be fused to other proteins or signal peptides to visualise the protein–protein interaction, protein movement in cytosol or individual organelles^42,54,55^. In particular, FPs differing in absorption and emission spectra are useful for cell painting when they are targeted at different organelles. A set of DNA vectors with various FP genes (cyan, green, yellow and red-mCherry) has been created. These genes were modified to express FPs fused to amino acid targeting sequences capable of bonding to and fluorescent labelling of various cellular organelles, including mitochondria, and they were used for organelle labelling^56^. To the best of our knowledge, until now, there have been no reports on the simultaneous use of different FPs, either for labelling protoplasts used as fusion components or for hybrid identification.

Breeding new F1 hybrid carrot cultivars relies on the use of male sterile lines. Male sterility in the carrot is determined by cytoplasm, thus a mitochondria transfer to a new genomic context is required for the development of new CMS lines. This is commonly done by repetitive backcrossing which is a difficult and long-lasting process, in particular in the carrot, an allogamous and biennial crop exhibiting strong inbreeding depression^57^. Protoplast hybridisation as an alternative approach may considerably shorten the time of cytoplasm transfer, however, its utilization requires the initial establishment of an efficient and reliable system confirming the successful transfer of mitochondria. Hence, the aim of this work was to develop a model allowing the confirmation of mitochondria transfer between protoplasts and the testing conditions favouring electrofusion. For this purpose, we developed a set of carrot lines whose cells had fluorescently labelled mitochondria by different FPs, and used them as sources of protoplasts for somatic hybridisation. We demonstrate that real-time observation of protoplast fluorescence during electrofusion enables the assessment of the effect of various DC parameters on protoplast stability and hybrid formation, and the optimisation of these parameters for somatic hybridisation. Moreover, for the first time, observations of the dual-colour fluorescence emitted by hybrid cells have directly confirmed a successful mitochondria transfer and enabled the estimation of the electrofusion efficiency in the carrot.

## Results and discussion

### Carrot cell transformation

Embryogenic cell suspensions of three varieties were used as convenient targets for genetic transformation ensuring effective exposure of plant cells to *A. tumefaciens*^58^. The microscopic observations of the ‘Amsterdamska’ suspension in the medium containing ammonium glufosinate conducted 3 days after transformation revealed the presence of elongated single cells and cell divisions. After 7 days, the suspension became denser and 1–3 mm micro clusters of cells were distinguished as was expected in the event of successful transformation^59^. Such changes in the ʻKoralʼ and DH suspensions were delayed, and cell aggregates were visible 12 days after transformation**.** Microscopic observations revealed that cell fractions resistant to ammonium glufosinate and capable of further divisions and development were present in all suspensions. The fluorescence observed 8–10 days after transformation was detected in all 12 cell suspensions (3 varieties × 4 FP genes). The non-transformed suspension cultured in the BI medium without a selection agent continued to grow but the cells did not emit fluorescence. Neither divisions nor fluorescence was found in the negative control, i.e. the non-transformed cell suspension treated with ammonium glufosinate.

The obtained suspension cultures consisted of non-fluorescing and fluorescing cells of various fluorescence intensities. Clear detection of fluorescence requires a high level of FP gene expression, which can be ensured, for example, by the 35S promoter, as in this work. It also requires the synthesis of FP peptide, which undergoes correct folding and maturation^60,61^. Disturbances in these processes may result in the lack of fluorescence or fluorescence quenching. A transient gene expression may also lead to unstable fluorescence. The models derived for carrot cells expressing GFP showed that weakening fluorescence was a function of time, which additionally depended on the carrot cultivar used as the cell source, and the fraction of fluorescing cells stabilised about a week after transformation^48^. For these reasons, the obtained suspensions in this work were composed of fluorescing cells, putatively transgenic cells not showing fluorescence and untransformed cells that survived a short period of selection. However, the identification of fluorescing cells indicated that the cell suspensions originating from the cells of the carrot storage root can be expedient for genetic transformation that had been questioned earlier^62,63^.

### Development of callus with FP-labelled cells

The occurrence of non-fluorescing cells in transgenic cell suspensions implied the need for a two-step selection using a solidified medium supplemented with the selection agent to develop homogenous lines of fluorescing cells. The formation of small aggregates on a solidified medium was observed first for the ʻAmsterdamskaʼ cells, and the process was noted just 5 days after the suspension transfer onto a filter paper disc placed on the medium surface. In the next 3 weeks, aggregates covered the filter paper, but then only some of them continued their growth and callus clumps became distinguishable. A similar development, although at a slower rate, was obtained for the cultures originating from the ʻKoralʼ and the DH line, which was congruent with the slower growth of these two varieties in the cell suspension culture, and confirmed the essential role of the source material used for transformation. Callus did not develop on the selection medium when non-transformed cells were cultured. The use of filter paper discs had been proposed earlier for an easy transfer of cells and their small aggregates to fresh nutrient media^59^, and was then adopted for selection purposes^64^. This procedure allowed repeated, undisturbed transfer of all cells and their exposure to the selection agent until the developing callus clumps could be separated and placed directly on the surface of fresh medium; hence, it increased the chance for the development of transgenic tissue. Four 6-week-old putative transgenic calli, growing well, from each treatment were sampled for PCR analysis. The amplification of the bar and FP gene fragments resulted in products of the expected lengths listed in Table 1 and confirmed the presence of each FP gene. Thus, molecular analyses provided evidence for successful gene transfer and selection of transgenic events in the callus.

**Table 1:** Primers used for polymerase chain reaction.

Primer	Sequence 5′–3′	Target gene	Expected product length
bar-F	ATGAGCCCAGAACGACGCCCGGCC	*bar*	409
bar-R	GCATGCGCACGGTCGGGTCGTTGG		
Fluo-F	TACATCAGCCACAACGTCTA	CFP, GFP, YFP	CFP—182 bp GFP and YFP—177 bp
Fluo-R	GACTGGGTGCTCAGGTAGT		
mCherry-F	CCGTAATGCAGAAGAAGACC	RFP	206 bp
mCherry-R	GGTGATGTCCAACTTGATGT		

**Figure 1:**
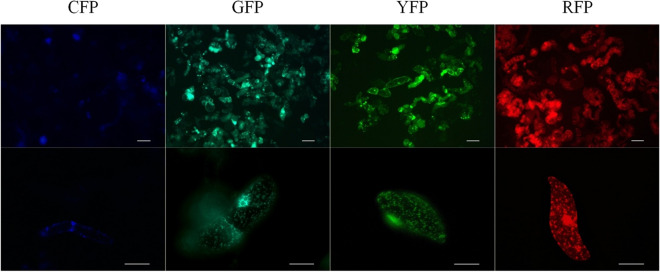
Fluorescence of carrot callus cells obtained after transformation with CFP, GFP, YFP and RFP plasmid vectors. Spread cells (upper row) and single cells at higher magnification (bottom row) with fluorescing mitochondria labelled with FPs and observed using dedicated excitation and emission filter sets. From the left: ‘Amsterdamska’-CFP, DH-GFP, DH-YFP, DH-RFP. Scale bar: 100 μm (upper row), 50 μm (bottom row).

All PCR positive callus lines emitted fluorescence; however, the proportion of fluorescing cells varied considerably among the materials and did not usually exceed 50% as estimated during a brief microscopic observation. At high magnification (400×), numerous, small, and scattered fluorescing spots were clearly identified within fluorescing cells (Fig. 1). The set of plasmid vectors used for transformation in this work contained a signal molecule targeting FPs to mitochondria and comes from the collection created by Nelson et al.^56^, who used it for fluorescent labelling of membrane-bounded organelles in *Arabidopsis* cells. Therefore, the fluorescing spots observed in cytoplasm could be identified as labelled mitochondria. The callus with introduced YFP emitted bright fluorescence, allowing for easy discrimination between fluorescing and non-fluorescing cells. Slightly lower fluorescence intensities were observed for GFP and RFP calli. Clear identification of single mitochondria tagged with CFP was not possible, as most CFP cells displayed faint fluorescence. The lower intensity of CFP fluorescence may be partially explained by the lower molar extinction coefficient and the lowest quantum yield of the ECFP variant in comparison to other FPs used. As a consequence, the ECFP brightness expressed in relation to the brightness of the reference EGFP is 39%, while for sGFP, EYFP and mCherry, the relative brightness values are 160%, 151% and 47%, respectively^65^. Despite the lower brightness, CFP has been useful in co-localisation studies using multicolour organelle labelling^66^.

The callus lines, including CFP, with the highest proportion of fluorescing cells and the highest fluorescence intensity were chosen for further culture and the second step of selection (Table 2). A brief inspection of fluorescence was conducted before every subculture to favour the transfer of callus fragments exhibiting the most intense fluorescence. In consequence, after the next 6 weeks of culture, the proportion of fluorescing cells in the developing calli increased (Table 3). However, in all CFP calli, CFP fluorescence remained faint; the scoring of fluorescing cells was ambiguous and clear discrimination between fluorescing and non-fluorescing cells was highly subjective. Hence, the CFP calli were excluded from further experiments despite their growth on the selection medium and positive molecular verification.

**Table 2:** Percentage of fluorescing cells in 6-week-old callus after *A. tumefaciens-*mediated carrot transformation with plasmid vectors harbouring FP genes.

Variety	Fluorescent protein tag	Number of cells	Estimated % of fluorescing cells
‘Amsterdamska’	CFP	172	85
	GFP	312	40
	YFP	248	40
	RFP	291	20
DH	GFP	261	50
	YFP	299	45
	RFP	207	60
‘Koral’	CFP	191	20
	GFP	213	25
	YFP	245	60
	RFP	253	35

**Table 3:** Mean protoplast diameter and percentage of fluorescing protoplasts isolated from 12-week-old callus.

Variety	Fluorescent protein tag	N	Mean diameter (μm)	N	Fluorescing protoplasts (%)
‘Amsterdamska’	GFP	736	25.9d ^1^	743	86.0c
	YFP	504	28.3c	1472	79.9d
	RFP	530	31.8b	900	60.4e
DH	GFP	718	22.7e	1082	87.6bc
	YFP	1026	22.3e	1409	81.5d
	RFP	414	27.0cd	1105	95.8a
‘Koral’	YFP	679	33.0a	944	90.1b

*N* number of protoplasts.

^1^Means followed by the same letter do not differ at *P* = 0.05 according to the Tukey’s test for protoplast diameter and Chi-square test for percentage of fluorescing protoplasts.

### Protoplast isolation from FP-labelled callus lines

Depending on the callus line, the isolation efficiency using a modified washing protocol was 1.2–2.6 × 10^6^ protoplasts per -gram of callus, which was in the range expected for a well working isolation procedure applied to tissue in a good physiological condition^6,67,68^. The obtained protoplasts were spherical, indicating complete enzymatic digestion of the cell wall and their diameter ranged from 22 to 33 μm depending on the line (Table 3). The cell size variation was noticeable among the callus lines originating from the same carrot variety but, on average, DH protoplasts (23.3 µm) were smaller (*P* < 0.001) than the ‘Amsterdamska’ and ‘Koral’ protoplasts (28.2 µm and 33.0 µm, respectively). The DH callus was obtained from the roots of the DH carrot line, which was bred through the reproduction of a doubled haploid plant achieved after doubling the haploid chromosome set^69^. In contrast to other varieties, the DH line is thus completely homozygous and characterised by a smaller leaf and storage root size that is common for carrot inbred lines, which usually exhibit strong inbreeding depression^57,70^. The smaller cell size of DH line may result from inbreeding depression due to complete homozygosity.

**Figure 2:**
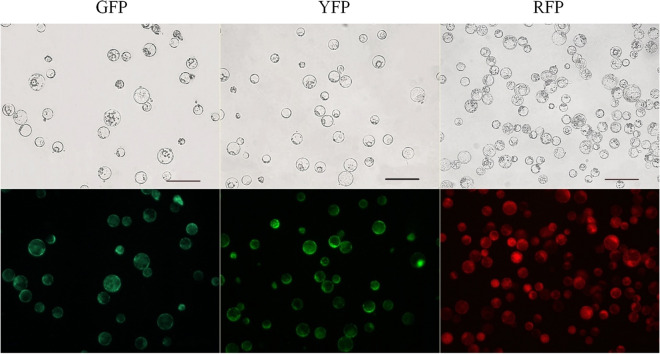
Protoplasts observed in bright field mode (upper row) and in reflected light (bottom row) after excitation with a mercury lamp using dedicated excitation and emission filter sets. From the left: DH-GFP, ‘Koral’-YFP, DH-mCherry. Scale bar of 50 μm is the same for the upper and bottom images.

The percentage of protoplasts emitting fluorescence was high (Fig. 2); in most 12-week-old callus lines, it was above 80% up to 95% (Table 3). These values were, on average, 2–3 times higher than the estimated percentages in a 6-week-old callus. Although direct comparison of fluorescence of isolated protoplasts in suspension and in callus cells should be done with caution, the obtained percentages indicate successful selection of fluorescing callus fragments done during consecutive subcultures and on the development of calli composed mainly of fluorescing cells. Among the ‘Koral’ callus lines, a high proportion of fluorescing cells was obtained only for the YFP line. The high homogeneity of the callus with regard to cell fluorescence made these lines valuable material as a source of protoplasts for further real-time observation of protoplast fusion. A lower fraction (60%) of fluorescing cells was observed only for the ‘Amsterdamska’ RFP callus, but fluorescence was bright, and such material was also used for protoplast fusion.

### Optimisation of electrofusion parameters

Real-time observations of the protoplasts isolated from the YFP-labelled ‘Koral’ callus allowed identification of distinguished electrofusion stages described earlier by Navrátilová^71^ and Hu et al.^28^. At first, the protoplasts were randomly scattered between two electrodes. Their dispersion occurred due to mutual repulsion of the negatively charged protoplast membranes until the alternating electric field was switched on. Then the adhesion of protoplasts forced by dielectrophoresis caused protoplast alignment in pearl chain structures. The application of DC caused reversible cell membrane perforation and, for some protoplasts, coalescence with the adhering protoplasts. Finally, after 3–5 s of membrane re-stabilisation, round hybrid protoplasts were formed. The hybrids of two cells had diameters larger than the donor protoplasts by about 25%, which was expected when the volume of a new hybrid protoplast was doubled.

**Figure 3:**
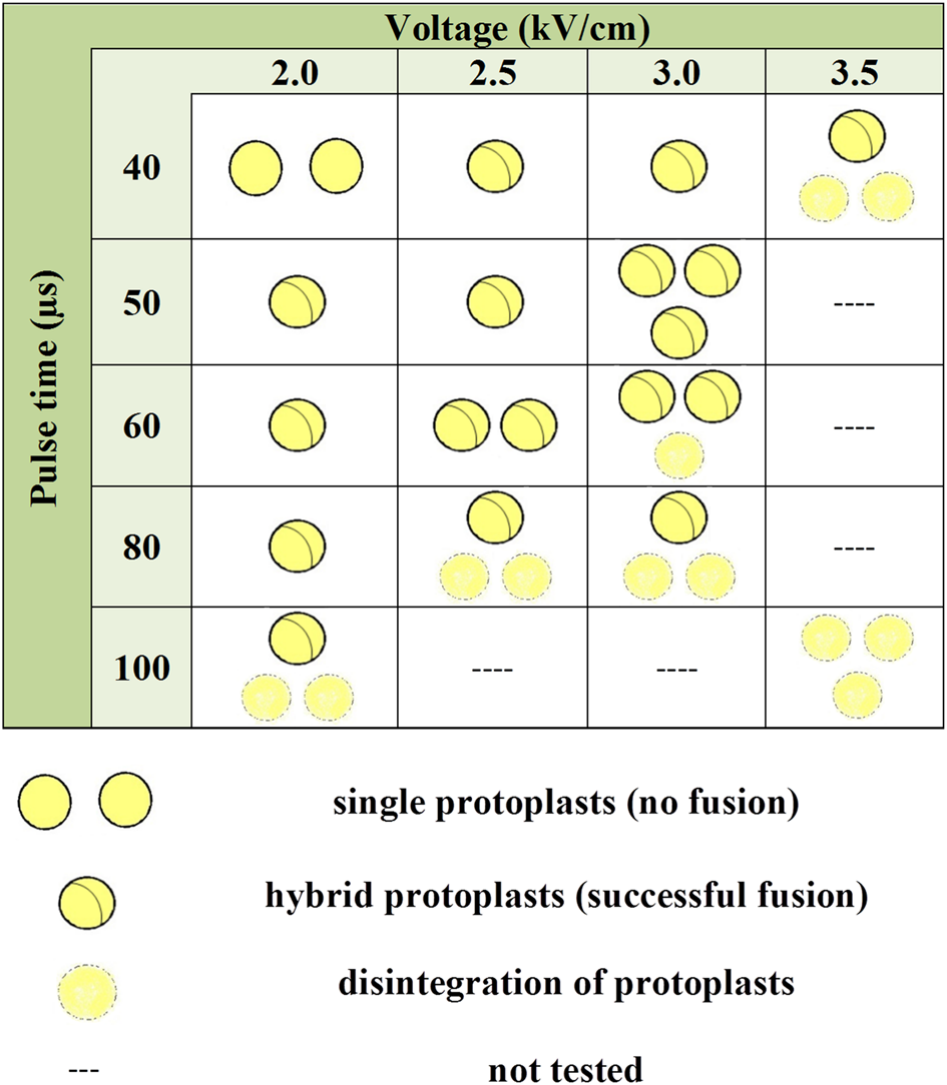
Schematic effect of DC voltage and electric pulse combinations on ‘Koral’ protoplast integrity and hybrid formation. The number of circles represents the relative frequency of hybrid or single protoplasts.

Membrane integrity and formation of hybrids highly depends on parameters of the applied electric field^32,72,73^ hence, the effect of fifteen combinations of DC voltages and pulse times on hybrid formation was assessed. In general, the cells remained intact, and no fusion was observed at low voltage and with short pulses (Fig. 3). Gradually increasing voltage above 2 kV/cm or pulse time above 40 μs led to fusion, but in most cases, the fusion was incomplete. At high values of these parameters, hybrids were formed but they were unstable and eventually disintegrated. Setting the parameters to the highest voltage (3.5 kV/cm) and longest pulse (100 μs) caused irreversible protoplast damage. Complete fusion and formation of stable hybrids was observed when applying intermediate voltage values (2.5 and 3.0 kV/cm) and pulse times (50 or 60 μs). These observations are consistent with the thesis that shorter pulses of higher voltage are preferable in successful protoplast electrofusion^74^. As the result of screening, these 4 out of 15 combinations of current parameters were considered to favour hybrid formation.

### Real-time identification of heterofusants

**Figure 4:**
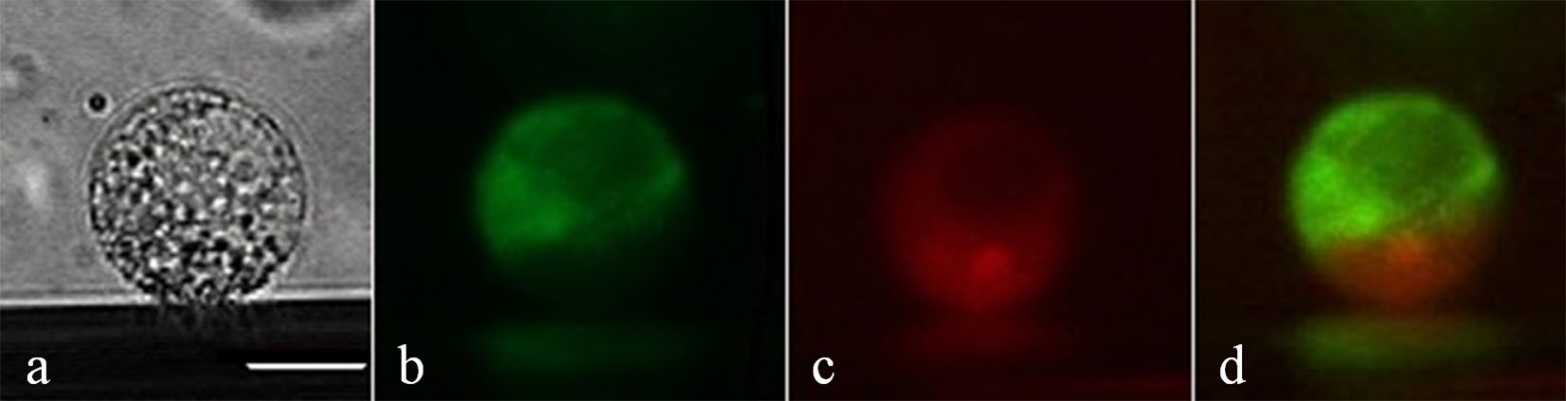
Identification of the heterofusants after DH-RFP and ‘Amsterdamska’ (A)-GFP protoplast fusion. Hybrid protoplasts were observed in transmitted light and in reflected light using excitation/emission filters matching FP used for labelling. Hybrid in transmitted light (**a**), hybrid emitting green fluorescence due to GFP present in A-GFP component protoplast (**b**), hybrid emitting red fluorescence due to RFP present in DH-RFP component protoplast (**c**), merged images using Image J2x v.2.1.4.7 computer software (https://imagej.net/ImageJ) showing dual-colour fluorescence emitted by both components in the heterofusant (**d**). Scale bar: 25 μm.

When somatic hybridisation aims to combine protoplasts that have different genetic backgrounds, the identification of heterofusants and distinguishing them from homofusants is crucial. In this work, the protoplasts were labelled with three FPs, GFP and YFP, whose spectra partially overlap^75^, and RFP which emits fluorescence in longer wavelengths^76^. Hence, two component combinations, RFP + GFP and RFP + YFP, were chosen to detect heterofusants. Real-time observations during electrofusion allowed the differentiation of protoplasts labelled with the FPs emitting either red or green/yellow fluorescence. After the electrofusion process was completed, it was possible to identify protoplasts emitting fluorescence in two spectra (Fig. 4). Observation of dual-colour fluorescence indicated that such protoplasts were composed of two fused components, each labelled with different FPs (RFP + GFP or RFP + YFP); hence, they were identified as heterofusants. An inspection in transmitted light was carried out to verify whether there were any other protoplasts in close proximity or whether there were two protoplasts that overlapped which could cause a similar visual effect, and to exclude the possibility of false identification.

### Fusion efficiency

**Figure 5:**
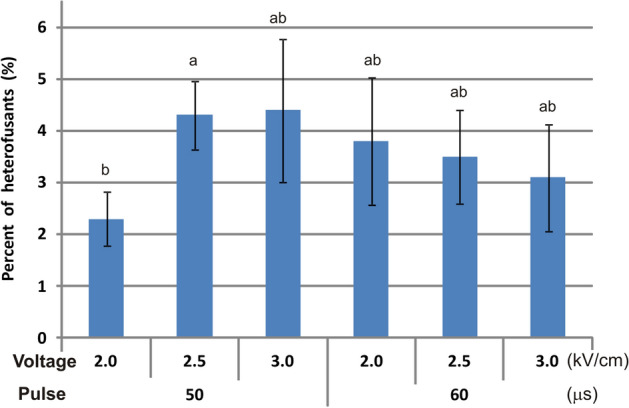
Mean efficiency (%) of heterofusant formation depending on DC voltage and pulse time during electrofusion. Means followed by the same letters do not differ significantly according to the Tukey’s test at *P* = 0.05; n = 16. Whiskers represent standard errors.

Eight pairs of callus lines were used for protoplast electrofusion and four combinations of voltage and pulse time were set, based on the above-described optimisation experiments carried out using ‘Koral’ protoplasts. Additionally, a lower DC of 2 kV/cm was applied for 50 and 60 µs to extend the range of electrofusion parameters including those that were considered less harmful. The percentage of obtained heterofusants ranged from 0.9 to 13.9%, but at the highest voltage (3.0 kV/cm) or a longer pulse (60 µs) the fusion was often unsuccessful (Table 4). The efficiency highly depended on the pair of fusion components (*P* < 0.001) and their interaction with DC voltage and pulse (*P* < 0.001); this 3-way interaction explained one third of total variation. Two-way interactions between fusion components and DC voltage or DC pulse explained another 26% of variation (both *P* < 0.001). On average, the efficiency doubled by increasing DC voltage from 2.0 to 2.5 kV/cm, but only when the pulse lasted 50 µs (Fig. 5). Further voltage increase to 3.0 kV/cm favoured heterofusant formation for two line combinations and was adverse for the remaining five combinations. At the longer pulse (60 µs), changes to voltage did not substantially affect the mean efficiencies.

**Table 4:** Estimated efficiency (%) of heterofusant formation after electrofusion of protoplasts isolated from carrot callus lines with cells labelled with different FPs depending on DC voltage and pulse time.

Fusion components		Pulse time (μs)	Voltage (kV/cm)								
			2.0			2.5			3.0		
1st	2nd		Mean	s.e		Mean	s.e		Mean	s.e	
A-RFP^a^	DH-GFP	50	2.9	0.91	g–m^1^	1.8	0.02	j–m	10.3	0.64	a–c
		60	5.3	0.23	c–k	9.0	0.37	a–e	0		n
	A-GFP	50	2.4	0.01	h–m	4.4	0.20	d–k	0		n
		60	0		n	0		n	0		n
	A-YFP	50	3.3	0.44	f–l	1.3	1.25	l–n	12.3	2.47	a–b
		60	0		n	3.5	3.54	j–m	0		n
	K-YFP	50	0		n	7.0	0.49	b–g	0		n
		60	4.4	1.78	e–k	3.2	0.70	g–m	9.9	2.99	a–d
DH-RFP	A-GFP	50	0.9	0.15	l–n	4.3	0.16	d–k	0		n
		60	6.6	0.53	b–h	0		n	4.7	1.72	d–k
	DH-GFP	50	6.1	2.40	c–i	5.6	0.65	c–j	9.9	3.31	a–d
		60	13.9	1.99	a	8.6	0.61	a–e	8.2	0.58	a–f
	DH-YFP	50	1.0	1.02	m–n	8.2	1.88	a–f	2.4	0.98	h–m
		60	0		n	1.5	0.25	k–m	0		n
	K-YFP	50	2.2	0.37	i–m	2.0	0.18	i–m	0		n
		60	0		n	2.1	0.42	i–m	1.7	0.08	j–m

*A* ‘Amsterdamska’, *K* ‘Koral’, *DH* DH1 line, *s.e.* standard error.

^1^Means followed by the same letters do not differ significantly according to the Tukey’s test at *P* = 0.05. Two fusion experiments were performed for each factor combination using protoplasts from independent isolations. At least 100 protoplasts were observed per each combination with the exception for DH-RFP + A-GFP and DH-GFP for which at least 30 cells were observed.

The combination of callus lines used as fusion components affected the heterofusion efficiency (*P* < 0.001) and highly contributed (30%) to total variation. The highest efficiency (8.7% ± 1.01 s.e.) observed for the DH-RFP + DH-GFP pair was eight times greater than for the A-RFP + A-GFP pair (1.1% ± 0.51 s.e.). On average, the combination of protoplasts isolated from two DH callus lines labelled with different FPs more often resulted in successful heterofusant formation (*P* = 0.005; n = 24), with a mean efficiency of 5.5% ± 0.9 s.e., than when the protoplasts were isolated from the two ‘Amsterdamska’ callus lines (2.3% ± 0.8 s.e.). The DH protoplasts were smaller than the ‘Amsterdamska’ protoplasts, however, the protoplast size did not explain the observed differences in the effectiveness of heterofusant formation. When protoplasts of similar size, 22.7 µm (DH-GFP) and 22.3 µm (DH-YFP), were used for fusion in combination with DH-RFP, the mean efficiencies (8.7% and 2.2%, respectively) differed significantly (*P* < 0.001; n = 12). Thus, the results do not support the conclusions from other reports^31^ that the use of protoplasts of similar size favours hybridisation and are congruent with those questioning such relationships and explaining the differences in fusion response with different metabolic characteristics of fused cells^72^. The DC voltage and DC pulse main effects were also statistically significant (*P* = 0.001 and *P* = 0.013, respectively) but their contribution to the total variation was only 3%.

Considering both the efficiency of heterofusant formation and the positive response of various materials to electrofusion, the DC parameters set to 2.5 kV/cm and 50 µs were the most effective. At these parameters, heterofusants were obtained in all experiments for all pairs of components, and the efficiency ranged from 1.3 to 8.2%, with a mean of 4.3% ± 0.65 s.e. To the best of our knowledge, the exact electrofusion parameters are given in only one report describing the development of cybrid carrot plants after fusion of suspension-derived protoplasts using two DC pulses of 50 µs at 1.0 kV/cm, however, the results concerning the efficiency were not presented^21^. The efficiency obtained in this work cannot be directly compared, as there is no such data available for the carrot. In other species, also with the help of different set of morphological markers, the estimation of heterofusants frequency is difficult and time consuming, and hence such results have been rarely presented. In Brasicaceae, the frequency of heterofusant formation after PEG- and electric field-induced fusion ranged from 0.5 to 10%^71^, while in the citrus species, electrofusion led to 1–5% of heterofusants^27^. Higher values (11–22%) were noted during electrofusion between pea and grass pea protoplasts^77^. The results presented in this work indicate that electrofusion of callus-derived carrot protoplast may lead to heterofusant formation with satisfactory efficiency in comparison to other species.

The efficiencies estimated here did not account for the fact that electrofusion might additionally lead to homofusion. Homofusants could not be identified by observation of dual-colour fluorescence, as they emitted fluorescence in only one spectrum range. An indirect confirmation of homofusant formation was the enlargement of some protoplasts after electrofusion and their fluorescence in one spectrum range only. Quantification of the frequency of this process would be, however, highly biased due to protoplast variation in size observed before fusion. Assuming the probability of homo- and heterofusion was similar, as the protoplasts of both components were mixed in a 1:1 ratio, the mean efficiency would be equal to a doubled frequency of heterofusant formation i.e., above 8%.

In general, electrofusion is considered advantageous over the PEG-mediated fusion due to its low cell toxicity, but the latter approach is preferably chosen, independent of the plant species^28^. Electrofusion requires the use of special equipment, such as pulse generator, and the optimisation of alternating current and DC parameters, which must be adjusted depending on the species, cell type and cell size. Papers describing the effect of all these factors are rare and they concern the electrofusion of protoplasts derived mainly from mesophyll and cell suspensions^27,72^. The reported ranges of applied DC parameters are wide. Mostly, DC voltages ranged from 1.0 to 3.5 kV/cm, pulses lasted for 40–100 µs, and DC was applied once or repeated 2–3 times, when using pairs of the same or different cell types as fusion components. These components included suspension/suspension- leaf/leaf- or suspension/callus-derived protoplasts^78–80^. In the present work, the selected DC parameters ensuring the highest efficiency of carrot hybrid formation fit in these ranges, although a shorter pulse time was more advantageous.

### Cell development in post-fusion mixture

**Figure 6:**
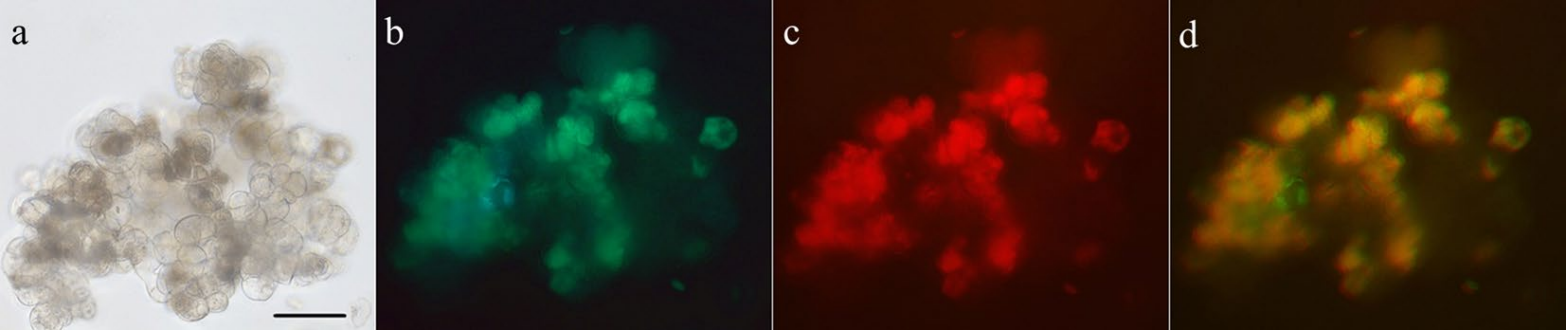
A 20-day-old hybrid cells aggregate after DH-GFP and DH-RFP protoplast fusion. The aggregate was observed in transmitted light and in reflected light using excitation/emission filters matching FP used for labelling. Cell aggregate in transmitted light (**a**), cells emitting green fluorescence (**b**), cells emitting red fluorescence (**c**), merged images using Image J2x v.2.1.4.7 computer software (https://imagej.net/ImageJ) showing dual-colour fluorescence emitted by hybrid cells of the aggregate (**d**). Scale bar: 100 μm.

The FDA staining revealed that the viability of DH-RFP and DH-GFP protoplasts before fusion was high (71% and 78%, respectively). After electrofusion at the DC parameters set to 2.5 kV/cm and 50 µs, the viability of protoplasts in the mixture remained at a similar level (71%). First, mitotic divisions were observed 5 days after electrofusion; then cells formed cell aggregates constituting 14.2% ± 1.0 s.e. and 16.6% ± 0.9 s.e. of all observed cells/aggregates in 7- and 10-day-old cultures, respectively. In 20-day-old cultures the percentage of cell aggregates increased 4–5-fold to 66.8 ± 1.8 s.e. (*P* < 0.001). Dual-colour fluorescence in heterofusants was visible both after the first mitotic division and in forming the cell aggregates (Fig. 6). The obtained results of cell viability and mitotic activity show that the chosen electric conditions had no adverse effect on carrot protoplast cultures. The available reports describing carrot cell behaviour in protoplast cultures present a similar level of cell viability before culture and a similar cell growth kinetics with respect to entering into the first mitotic division as well as formation of cell aggregates^6,68,81,82^.

## Conclusions

The presented results have shown that carrot protoplasts can be stably labelled by FPs targeting mitochondria and, for the first time, that such fluorescent markers can be useful for immediate discrimination of parental components during the whole process of protoplast fusion, stimulated by the application of electric current. They have also demonstrated that the hybrids can be identified due to the emission of dual-colour fluorescence. Despite avoiding the need for additional protoplast staining, the protoplast response to applied conditions varied greatly, indicating that the physiological condition of fusion components remains one of the most critical factors in somatic hybridisation. Moreover, the optimal conditions favouring fusion for one pair of components could not be as good as for another pair, and there was no direct relationship between hybridisation efficiency and protoplast size or protoplast origin. Nonetheless, some combinations of DC parameters ensured successful fusion of any pair of components and the obtained hybrid cells underwent divisions; hence, they should be selected for further somatic hybridisation in the carrot using other protoplast sources.

## Material and methods

### Biological material

Well established, 4–6-month-old, embryogenic cell suspensions derived from storage roots of three carrot (*Daucus carota* L. ssp. *sativus* Hoffm.) varieties were used: a doubled haploid DH1 line (DH)^69^ and two cultivars: Amsterdamska (A) and Koral (K). The cell suspensions were maintained in a liquid BI mineral medium composed of Gamborg B5 salts with vitamins (Duchefa, Haarlem, the Netherlands) supplemented with 1 mg/L 2,4-dichlorophenoxyacetic acid (2,4-D) (Sigma, St. Louis, USA), 0.0215 mg/L kinetin (Sigma), 30 g/L sucrose, pH 5.8, and incubated using a gyratory shaker (250 rpm) at 26 °C in the dark. The subcultures were done every 2 weeks by transferring 5 mL of suspension into 15 mL of fresh liquid medium.

### Plasmid vectors

A set of four pFGC19 plasmid vectors (mt-cb CD3-986, mt-gb CD3-988, mt-yb CD3-990 and mt-rb CD3-992^56^ was obtained from Arabidopsis Biological Resource Center. The T-DNA region of each vector contained the ammonium glufosinate resistance *bar* gene driven by mannopine synthase promoter and one of the following fluorescence protein (FP) genes under a double 35S promoter control: enhanced cyan FP (*eCFP*), S65T variant of green FP (*sGFP*), enhanced yellow FP (*eYFP*) and the *mCherry* variant of red FP^83^, hereinafter denoted in this paper as CFP, GFP, YFP and RFP, respectively. All FP gene sequences were preceded by a mitochondrial targeting sequence of the first 29 amino acids of *Saccharomyces cerevisiae* cytochrome *c* oxidase IV—ScCOX4^84^. Plasmids were transferred into the LBA 4404 *Agrobacterium tumefaciens* strain by electroporation^85^.

### Carrot transformation and selection

*Agrobacterium tumefaciens* was cultured in lysogeny broth (LB) with 50 mg/L kanamycin on a gyratory shaker (250 rpm) at 26 °C. An overnight culture was centrifuged for 10 min, and the pellet was re-suspended in 1 mL of BI medium, and then the inoculum was diluted to OD_600_ = 0.5. The inoculum (100 μL) was added to 20 mL of the carrot cell suspension containing 20 μL of 100 μM acetosyringone. After a 48-h incubation (250 rpm, 26 °C, in the dark), 200 mg/L of timentin and 400 mg/L of cefotaxime were added to kill the bacteria. Then, the suspension was transferred in 1-mL aliquots on the surface of sterile filter paper disks placed on a 0.27% Phytagel (Sigma) solidified BI medium in Petri dishes supplemented with 200 mg/L cefotaxime, 100 mg/L timentin and 10 mg/L ammonium glufosinate as a selection agent. The filter paper discs were transferred to fresh media every 2 weeks, and then the developing, individual callus clumps were directly placed on BI selection medium. The callus was cultured at 26 °C in the dark and was subcultured every 2 weeks to a fresh medium with the addition of 10 mg/L ammonium glufosinate. The callus clumps obtained from the non-transformed carrot cell suspensions were considered as negative controls. The remaining part of the suspension not transferred to Petri dishes was further cultured on the gyratory shaker after supplementation with 5 mg/L ammonium glufosinate.

### PCR

Genomic DNA was isolated from 6-week-old callus using the CTAB method according to the protocol by Rogers and Bendich^86^ with modifications as described by Klimek-Chodacka et al.^87^. The PCR reaction was conducted in a Mastercycler gradient thermocycler (Eppendorf, Hamburg, Germany) under the following conditions: the initial denaturation for 3 min at 94 °C, 30 cycles of 30 s at 94 °C, 30 s at 56 °C and 30 s at 72 °C, followed by the final extension for 3 min at 72 °C. The 10 μL of reaction mixture included 50 ng/μL DNA, 0.1 μM of each primer (Table 1) and 2 × buffer including *Taq* polymerase and dNTPs (PCR Mix Plus, A&A Biotechnology, Gdynia, Poland). The amplified products were visualised using the MidoriGreen Advance dye (Nippon Genetics) after electrophoresis in 1.5% agarose gel.

### Protoplast isolation

Protoplasts were isolated from FP-labelled carrot callus after growing for 2 weeks after the last subculture according to the protocol by Grzebelus et al.^68^ with minor modifications. The protoplasts obtained after gradient centrifugation in W5 and sucrose/MES solutions were washed three times in a chilled 0.4 M mannitol solution and centrifuged at 100 × *g* for 5 min after each washing. Beginning from the first washing, the protoplasts were kept on ice. The final density of the protoplast suspension was adjusted with 0.4 M mannitol solution to 2 × 10^5^ protoplasts per millilitre as determined by counting using the Fuchs–Rosenthal haemocytometer chamber.

### Protoplast fusion

Protoplast fusion was carried out using the Multiporator Electroporation Systems (Eppendorf, Hamburg, Germany) designed for electroporation of eukaryotic cells. First, 30 μL of protoplast suspension were placed into a micro-fusion chamber dedicated for microscopic observations that had two electrodes at a 200-μm distance. To perform the fusion between the protoplasts labelled with different FPs, equal volumes of both fusion components were gently mixed before application. The micro-fusion chamber was mounted on a microscope table of the inverted Zeiss AxioObserver A1 microscope (Carl Zeiss, Göttingen, Germany) with fluorescence mode and connected to the Multiporator through a dedicated coaxial cable. To force a pearl chain alignment of protoplasts, an alternating current of 175 V/cm was applied for 15 s. Then, to destabilise the membranes and fuse the protoplasts, various combinations of direct current were tested. The voltage was set to 2.0, 2.5, 3.0 or 3.5 kV/cm during one electric pulse set to 40, 50, 60, 80 or 100 μs. Then, the final alternating current of 50 V/cm was applied for 5 s to re-stabilise protoplasts. The effect of fusion parameter combinations on hybrid formation was assessed using the protoplasts of the ʻKoralʼ callus. The selected combinations were applied to the mixtures of protoplasts derived from two callus lines with cells labelled with different FPs.

### Monitoring of the post-fusion cell development

After DH-RFP and DH-GFP protoplasts electrofusion at the selected optimal DC conditions, the protoplasts were embedded in thin alginate layers and cultured in the protoplast culture medium according to a previously described protocol by Grzebelus et al.^68^. The protoplast viability before and just after electrofusion was verified by fluorescein diacetate (FDA) staining^68^. Additionally, the formation of cell aggregates was observed in 7-, 10- and 20-day-old cultures, and the percentage of cell aggregates was determined in relation to the total number of observed cells/aggregates.

### Microscopy

Aliquots of cell suspensions or protoplasts were directly placed on a microscopic slide. Pieces of callus were submerged in distilled water first, and then aliquots of loosened cells were transferred to a microscopic slide. The preparations were examined using the inverted Zeiss AxioObserver A1 microscope with fluorescence mode (Carl Zeiss) equipped with a set of High Efficient (HE) filters dedicated to observation of fluorescence. For the FP variants, the following filters were used: CFP—HE47 (excitation wavelength λ_Ex_ = 436/25, emission wavelength λ_Em_ = 480/40), GFP—HE38 (λ_Ex_ = 470/40, λ_Em_ = 525/5), YFP—HE46 (λ_Ex_ = 500/25, λ_Em_ = 535/30) and RFP—HE63 (λ_Ex_ = 572/25, λ_Em_ = 629/62). The cells and protoplasts were observed in bright field mode using 200× magnification. During electroporation, the protoplasts were observed in transmitted light (bright field mode) and in reflected light (fluorescence mode) after excitation with a mercury lamp. Each specimen was observed using HE filters changed according to the FP present in the cells. The images were captured using the AxioCam MRc 5 camera (Carl Zeiss) attached to the microscope. To observe a mixture of protoplasts possessing mitochondria labelled with different FPs and to detect hybrid protoplasts emitting dual-colour fluorescence after fusion, the recorded images taken using different HE filters were merged using ImageJ2 × 2.1.4.7 software (https://imagej.net/ImageJ). Green fluorescence of live cells after FDA staining was examined using filter set HE38 (λ_Ex_ = 470/40, λ_Em_ = 525/5).

### Statistical analysis

The diameters of 400–1000 protoplasts per callus line were measured using AxioVision v.4.8 (Carl Zeiss MicroImaging) software. The data were subjected to a one-way analysis of variance (ANOVA) and the Tukey’s test was used for a pairwise mean comparison at *P* = 0.05. Homogenity of fluorescence was described by counting fluorescing callus cells (170–300 cells per line) or isolated protoplasts (740–1470 per line) and expressed as the percentage of all visible cells/protoplasts in transmitting light. The Chi-square test with Yates correction was used to identify significant differences between proportions of fluorescing protoplasts. After electrofusion, the efficiency of heterofusant formation was estimated by calculating the percentages of protoplasts emitting dual-colour fluorescence and dividing these percentages by the proportions of fluorescing protoplasts determined for fusion components before electrofusion. This correction was applied to take into account for fluorescence heterogeneity in source materials. A three-factor ANOVA (pair of fusion components, DC voltage and DC pulse time) was performed after Bliss transformation of percentage data. Significance between means was verified using the Tukey’s test. Two fusion experiments were performed for each factor combination using protoplasts from independent isolations (96 fusions in total) and at least 100 cells per combination were observed. The assessment of protoplast viability and colony formation in cell culture after electrofusion at the optimal DC conditions was carried out on 200–350 cells per each time point in four repetitions and data were subjected to one-way ANOVA followed by the Tukey’s test.

## Abbreviations

CFP: Cyan fluorescent protein
DC: Direct current
FP: Fluorescent protein
GFP: Green fluorescent protein
RFP: Red fluorescent protein (mCherry variant)
YFP: Yellow fluorescent protein
